# The correlation between heavy metal ions in blood and metabolic dysfunction-associated steatotic liver disease from 1999 to 2018 based on NHANES data

**DOI:** 10.3389/fpubh.2024.1512901

**Published:** 2025-01-07

**Authors:** Haijun Ma, Jun Zhao, Jian Xu

**Affiliations:** 1 Department of General Surgery, The First Affiliated Hospital, Jiamusi University, Jiamusi, China; 2 Department of Blood Transfusion, The First Affiliated Hospital, Jiamusi University, Jiamusi, China

**Keywords:** metabolic dysfunction-associated steatotic liver disease (MASLD), NHANES, blood cadmium, blood mercury, liver disease, metabolic dysfunction

## Abstract

**Background:**

Metabolic-associated steatohepatitis and liver fibrosis (MASLD) is a growing public health concern, with environmental factors potentially playing a role in its development. This study aimed to investigate the associations between serum cadmium and mercury levels and the risk of MASLD in a nationally representative sample from the United States.

**Methods:**

Data from the National Health and Nutrition Examination Survey from 1999 to 2018 were analyzed. Serum cadmium and mercury concentrations were measured, and MASLD was defined based on established criteria. Logistic regression models were used to assess the associations between serum metal levels and MASLD, with adjustments for potential confounders. Stratified analyses and restricted cubic spline curves were employed to examine subgroup differences and nonlinear relationships.

**Results:**

The study revealed significant inverse associations between serum cadmium and mercury levels and the likelihood of MASLD. Individuals in the highest quartiles of cadmium and mercury had lower odds of MASLD compared to those in the lowest quartiles (Model 3: Cadmium Q4 vs. Q1, Mercury Q4 vs. Q1). Stratified analyses showed stronger inverse associations in older adults, males, and never smokers for cadmium, and in females and individuals without diabetes for mercury. Nonlinear dose–response curves indicated critical thresholds beyond which the risk dynamics changed.

**Conclusion:**

Higher serum levels of cadmium and mercury were associated with a lower risk of MASLD, with notable variations across subgroups. These findings challenge the conventional understanding of these heavy metals as universally harmful and highlight the need for further research to unravel the complex interplay between environmental exposures and MASLD pathophysiology.

## 1 Introduction

Metabolic Dysfunction Associated Steatotic Liver Disease (MASLD) represents a pivotal shift in the understanding and categorization of liver diseases related to metabolic dysregulation. This condition, formerly recognized within the spectrum of Non-Alcoholic Fatty Liver Disease (NAFLD), emphasizes the crucial role of metabolic dysfunction in the pathogenesis and progression of liver steatosis absent of significant alcohol consumption. The reclassification to MASLD is not merely terminological but is intended to encapsulate a broader and more precise spectrum of etiological and pathophysiological features that align more closely with metabolic syndrome components such as insulin resistance, obesity, and dyslipidemia (1–3). This condition has emerged as a significant global health concern due to its potential progression to cirrhosis, hepatocellular carcinoma, and liver failure (4–9). Recent studies have suggested a possible link between environmental factors, particularly the exposure to certain heavy metals, and the prevalence of MASLD. Heavy metal pollution in the blood may exacerbates MASLD by increasing oxidative stress, which intensifies lipid peroxidation and hepatocyte damage (10). Metals like arsenic enhance inflammatory responses, advancing steatosis to steatohepatitis (11). Disrupted metabolic regulation from impaired endocrine functions promotes insulin resistance, crucial in MASLD progression. Additionally, chronic exposure to heavy metals stimulates hepatic stellate cells (10), accelerating fibrosis and worsening liver pathology, potentially leading to cirrhosis and hepatocellular carcinoma (12, 13). Building on the insights from recent studies that delineate the harmful effects of heavy metals on liver health, particularly their role in exacerbating the pathological processes underlying MASLD, there is a compelling need to further investigate these associations. Specifically, the influence of heavy metals such as mercury, cadmium, and lead on the progression of MASLD remains poorly understood. Given the potential severity of MASLD outcomes, including steatohepatitis, fibrosis, and even hepatocellular carcinoma, a deeper exploration into the quantitative impact of these metals is essential.

The choice of heavy metals for this study is predicated on their well-documented roles as environmental toxins with the ability to disrupt metabolic processes (14–16). Mercury, cadmium, and lead are ubiquitous in the environment and can enter the human body through various pathways, including food consumption, air inhalation, and occupational exposure (17). Once absorbed, these metals can exert toxic effects on liver cells, potentially contributing to or exacerbating liver dysfunction (18, 19). The liver’s role in detoxification processes makes it particularly vulnerable to these toxic insults, which may manifest as altered lipid metabolism and subsequent fat accumulation.

By leveraging the comprehensive and nationally representative National Health and Nutrition Examination Survey (NHANES) database, this study provides a unique opportunity to assess the correlation between blood levels of these metals and MASLD in a large, diverse population. The NHANES data allows for the control of various confounding factors, including demographic variables, lifestyle choices, and co-existing medical conditions, thereby offering a more nuanced understanding of the impact of these heavy metals on liver health.

This investigation not only contributes to the existing body of knowledge regarding environmental factors in MASLD pathogenesis but also may inform public health strategies aimed at reducing exposure to these potentially harmful metals. Through detailed statistical analysis of the NHANES data, this paper seeks to delineate the specific roles that mercury, cadmium, and lead may play in the development and progression of MASLD, thereby adding a crucial dimension to the ongoing discussion on environmental health and chronic liver diseases. This paper aims to explore the association between the concentrations of specific heavy metals—mercury, cadmium, and lead—in the bloodstream and the incidence of MASLD, utilizing data from the National Health and Nutrition Examination Survey from 1999–2018.

## 2 Materials and methods

### 2.1 Participants

**Figure 1:**
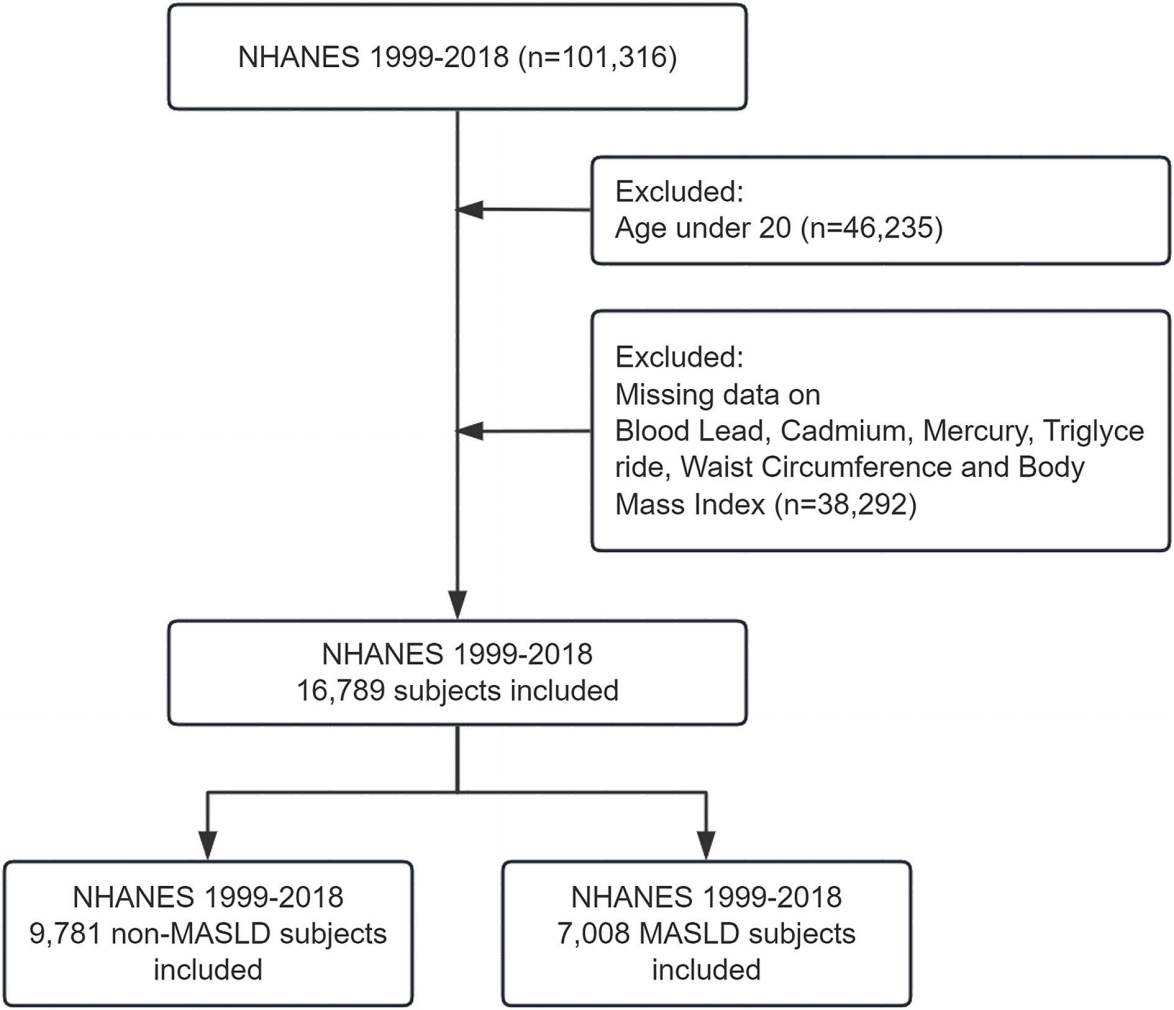
Flowchart of participant inclusion and exclusion criteria. Flowchart illustrating the inclusion and exclusion of participants from the NHANES 1999–2018 dataset. A total of 101,316 participants were initially included. After excluding individuals under 20 years of age (*n* = 46,235) and those with missing data on blood lead, cadmium, mercury, triglycerides, waist circumference, and body mass index (*n* = 38,292), 16,789 participants were eligible for the final analysis.

The present study harnessed data from the National Health and Nutrition Examination Survey to investigate the impact of blood concentrations of heavy metal ions (lead, chromium, and mercury) on the incidence of Metabolic dysfunction-associated steatotic liver disease. Utilizing data spanning from 1999 to 2018, the study incorporated participants who met stringent inclusion criteria. To qualify for inclusion, participants were required to be aged 20 years or older, ensuring that all subjects were adults, thus providing a more consistent basis for evaluating the effects of heavy metal exposure on metabolic health. Utilizing data spanning from 1999 to 2018, the study initially included 101,316 participants before exclusions. However, after a meticulous exclusion process that removed individuals with incomplete data sets—specifically those lacking comprehensive records on heavy metal concentration, liver health status, or other essential health metrics—a total of 16,789 participants remained eligible for analysis (Figure 1).

### 2.2 Definition of MASLD

For the purposes of this study MASLD was rigorously defined based on a combination of diagnostic criteria that integrated hepatic and metabolic dysfunction indicators. In the absence of direct ultrasonographic assessments of hepatic steatosis during most interview cycles, hepatic steatosis was determined using the Fatty Liver Index (FLI). The FLI is recognized as a reliable tool for evaluating steatotic liver disease (SLD), providing a non-invasive, indirect measure based on algorithms that incorporate various metabolic and anthropometric parameters (20, 21). An FLI score of 60 or higher was employed as a threshold to qualitatively signify the presence of SLD (20, 21). The FLI equation is listed below (22):

$$\mathrm{FLI}=100\times \frac{e^{\begin{array}{l} 0.953\times \mathrm{Ln}(\mathrm{TG})+0.139\times \mathrm{BMI}+0.718\times \\ \mathrm{Ln}(\mathrm{GGT})+0.053\times \mathrm{WC}-15.745 \end{array}}}{1+e^{\begin{array}{l} 0.953\times \mathrm{Ln}(\mathrm{TG})+0.139\times \mathrm{BMI}+0.718\times \\ \mathrm{Ln}(\mathrm{GGT})+0.053\times \mathrm{WC}-15.745 \end{array}}}$$

where TG = triglycerides, GGT = gamma-glutamyl transferase, BMI = body mass index, and WC = waist circumference.

Building upon the identification of SLD, the diagnosis of MASLD required the presence of additional metabolic abnormalities (20, 23, 24). The criteria for MASLD included either of the following: a BMI of 25 kg/m^2^ or greater for males, or aWC of 94 cm or greater; for females, a WC of 80 cm or greater was indicative. Furthermore, metabolic dysfunction was defined by one or more of the following parameters: a fasting blood glucose (FBG) level of 100 mg/dL or higher, a 2-h post-load glucose level of 140 mg/dL or higher, a hemoglobin A1c level of 5.7% or higher, the presence of diagnosed diabetes mellitus (DM), or ongoing treatment to lower blood glucose levels. Blood pressure criteria were also included, with MASLD being indicated by a blood pressure of 130/85 mmHg or higher or the receipt of antihypertensive medication. Additionally, the lipid profile was considered in the diagnosis. A fasting plasma triglyceride level of 150 mg/dL or higher, or treatment for lipid reduction, was required. For the delineation of abnormal lipid levels specific to gender, males with a plasma HDL-cholesterol level below 40 mg/dL and females with a level below 50 mg/dL, or those receiving lipid-lowering treatment, were considered to have met the criteria for MASLD.

### 2.3 Demographic characteristics and covariates

The demographic and clinical characteristics of participants were thoroughly recorded and analyzed to provide context for the assessment of the associations between heavy metal exposures and MASLD. The demographic data encompassed age, sex, annual family income, marital status, race, education level, residential instability (RIP), and lifestyle factors such as smoking and drinking habits. Health-related variables included the prevalence of hypertension, obesity, and diabetes within the cohort.

For each participant, the median and interquartile range (IQR) of blood concentrations of lead, cadmium, and total mercury were calculated to understand the distribution of these heavy metal exposures within the population. Similarly, the mean and standard deviation (SD) were computed for age and body mass index (BMI) to provide insights into the overall health profile of the participants.

Sex was reported as a percentage to reflect the gender distribution within the study population. Moreover, percentages were also used to detail the marital status, race, level of education, residential instability, smoking status, drinking behavior, and the prevalence of hypertension, obesity, and diabetes.

### 2.4 Concentration determination

Specimens for metal ion analysis were collected using the NHANES Mobile Examination Centers (MECs), which are strategically deployed across diverse geographic locations to ensure a representative sampling of the U.S. population. Upon collection, these specimens were immediately frozen at −30°C. The frozen samples were subsequently transported to the Centers for Disease Control and Prevention (CDC) laboratories in California for detailed analysis. During the sample preparation phase for mass spectrometry, blood samples were first agitated to ensure uniform distribution of cellular elements, crucial as metals like lead primarily bind to red blood cells. Samples with clots were excluded to maintain consistency. For dilution, each blood sample was mixed with equal parts water and 48 parts of a special diluent containing reagents that release metals from red blood cells, enhancing ionization and preventing instrument clogging. This mixture was then processed through inductively-coupled plasma to convert the liquid into aerosol, vaporize, atomize, and ionize the sample. Ions, along with argon gas, were channeled into a mass spectrometer where a Dynamic Reaction Cell (DRC) was used to either remove interferences or enhance ion signals. The ions passed through the DRC were selectively charged and directed toward an analytical quadrupole, and the ion impact was detected, converted to digital data, and analyzed to determine elemental concentrations. In our study, the Limit of Quantitation (LOQ) for each heavy metal ion analyzed was determined based on the sensitivity of the analytical instrumentation used. The LOQ values are critical for ensuring that our measurements are both accurate and reliable. Specifically, the LOQs were established at 0.25 μg/dL for lead, 0.16 μg/L for cadmium, and 0.16 μg/L for total mercury. In instances where metal concentrations fell below the limit of detection (LOD), a statistical imputation technique was employed to address these non-detectable values. Specifically, values below the LOD were substituted with the value of LOD divided by the square root of two (LOD/√2). For additional details on laboratory procedures and quality assurance measures, interested parties are encouraged to refer to the NHANES website.

### 2.5 Statistical analysis

All statistical analyses conducted within this study were performed using the R programming language. To account for the survey’s complex sampling design, all analyses incorporated sample weights, clustering, and stratification, ensuring that the results are representative of the U.S. civilian non-institutionalized population. To facilitate the comparison of baseline characteristics across different groups, the “tableone” package was utilized. The primary methodological approach for analyzing the data involved “survey” package in R, which facilitates generalized linear modeling for survey data. The analysis incorporated various models to account for potential confounders based on demographic and clinical data. Model 1 served as the baseline, with no adjustments. Model 2 was adjusted for age, gender, marital status, and race. Model 3 included additional adjustments for education, RIP, smoking status, and drinking behavior, providing a more comprehensive control for factors that could influence the study’s outcomes. These covariates were included in the models to isolate the effect of heavy metal concentrations on the incidence of MASLD from other influencing factors. To further elucidate the impact of chromium and mercury concentrations on the incidence of MASLD, stratified subgroup analyses were conducted. The stratification was based on several key variables identified as important modifiers of the relationship between heavy metal exposure and health outcomes. These included age, sex, body mass index, diabetes status, hypertension status, smoking status, and drinking status. The significance of the associations was determined using multivariate logistic regression, with results reported as odds ratios (ORs) with 95% confidence intervals (CIs). To explore potential effect modifications between heavy metal concentrations and subgroup variables, interaction tests were conducted. Interaction terms were incorporated into the multivariate logistic regression models as cross-product terms between heavy metal concentrations and subgroup variables. The significance of these interaction terms was assessed to determine whether the associations between heavy metals and MASLD differed across subgroups. Restricted cubic spline (RCS) was employed to model the non-linear relationship between heavy metal concentration and the probability of MASLD. This methodological approach, facilitated by the RMS package in R. *p*-value of less than 0.05 was considered statistically significant.

## 3 Results

### 3.1 Characteristics of participants

The study utilized data sourced from the National Health and Nutrition Examination Survey spanning from 1999 to 2018, focusing on the comparison between individuals diagnosed with MASLD and those without this condition. This comprehensive analysis included a wide array of biomarkers, sociodemographic characteristics, and health-related behaviors (Table 1).

**Table 1:** Characteristics of participants.

	Level	Non MASLD	MASLD	*p*
*n*		9,781	7,467	
Lead (ug/dL)*		1.17 [0.74, 1.80]	1.14 [0.73, 1.80]	0.373
Cadmium (ug/L)*		0.32 [0.20, 0.60]	0.30 [0.18, 0.53]	<0.001
Mercury (ug/L)*		0.96 [0.48, 2.00]	0.81 [0.44, 1.53]	<0.001
Age^a^		44.60 (17.14)	48.99 (15.54)	<0.001
BMI^a^		24.77 (3.50)	34.66 (6.06)	<0.001
Sex (%)	Female	5,756 (60.4)	3,471 (47.2)	<0.001
	Male	4,025 (39.6)	3,537 (52.8)	
Annual family income^a^		8.63 (9.82)	8.52 (9.96)	0.686
Marriage (%)	Married or live with parents	5,863 (63.3)	4,341 (65.6)	0.05
	Widowed/Divorced/Separated/Unmarried	3,918 (36.7)	2,667 (34.4)	
Race (%)	Mexican American	1,479 (7.3)	1,401 (10.1)	<0.001
	Non-Hispanic Black	1902 (10.3)	1,520 (11.8)	
	Non-Hispanic White	4,373 (68.5)	3,038 (67.6)	
	Other Hispanic	843 (5.6)	630 (5.4)	
	Other Race	1,184 (8.4)	419 (5.1)	
Education (%)	Colloage or higher	5,297 (62.4)	3,242 (54.3)	<0.001
	High school or lower	4,470 (37.5)	3,763 (45.6)	
	Missing data	14 (0.1)	3 (0.0)	
PIR (%)	Low	2,612 (20.1)	2,101 (22.4)	<0.001
	Mid	3,426 (35.9)	2,533 (38.3)	
	High	2,908 (44.1)	1810 (39.4)	
Smoke (%)	Current	2041 (21.5)	1,403 (20.0)	<0.001
	Ever	2085 (22.1)	2012 (28.9)	
	Never	5,655 (56.4)	3,593 (51.0)	
Drink (%)	> = 5 times/week	1,171 (12.9)	1,104 (16.3)	<0.001
	2–4 times/week	2,673 (31.5)	1,698 (27.9)	
	Once or less	5,937 (55.7)	4,206 (55.8)	
Hypertension (%)	Hypertension	3,049 (26.2)	3,972 (53.9)	<0.001
	Without hypertension	6,732 (73.8)	3,036 (46.1)	
Obesity (%)	Obesity	744 (6.9)	5,555 (80.0)	<0.001
	Without obesity	9,037 (93.1)	1,453 (20.0)	
Diabetes (%)	Diabetes	882 (5.9)	1,947 (22.2)	<0.001
	Without diabetes	8,899 (94.1)	5,061 (77.8)	
Waist Circumference (cm) ^a^		88.00 (9.71)	113.31 (12.57)	<0.001
Fasting Glucose (mmol/L) ^a^		5.52 (1.27)	6.38 (2.11)	<0.001
Two Hour Glucose (OGTT) (mmol/L) ^a^		5.85 (2.19)	7.40 (3.14)	<0.001
Glycohemoglobin (%) ^a^		5.38 (0.66)	5.85 (1.12)	<0.001
Triglycerides (mmol/L) ^a^		1.06 (0.55)	1.97 (1.54)	<0.001
Direct HDL-Cholesterol (mg/dL) ^a^		59.70 (16.71)	46.92 (12.51)	<0.001
Systolic Blood pressure (mmHg) *		116.00 [106.00, 126.00]	124.00 [114.00, 134.00]	<0.001
Diastolic Blood pressure (mmHg) *		68.00 [62.00, 76.00]	74.00 [66.00, 80.00]	<0.001

^*^Median [Interquartile Range]; ^a^Mean (Standard Deviation).

OGTT, Oral Glucose Tolerance Test; HDL, High-Density Lipoprotein; PIR, Poverty Income Ratio; BMI, Body Mass Index.

In terms of biomarker levels, median concentrations of lead and total mercury were slightly lower in the MASLD group compared to the non-MASLD group, though these differences did not reach statistical significance. Conversely, cadmium levels were significantly lower in the MASLD group (median [IQR]: 0.30 [0.18, 0.53] μg/L) compared to the non-MASLD group (0.32 [0.20, 0.60] μg/L), with a *p*-value of less than 0.001, indicating a substantial disparity.

Physiological measurements revealed notable differences between the groups. The mean age of the MASLD group was significantly higher at 48.99 years compared to 44.60 years in the non-MASLD group. Body mass index (BMI) also differed significantly, with the MASLD group presenting a considerably higher mean BMI of 34.66 compared to 24.77 in the non-MASLD cohort.

Sociodemographic data indicated that the MASLD group had a higher proportion of males and individuals living with parents or married. Although annual family income did not show significant differences between the groups, educational attainment did; a lower percentage of the MASLD group had college or higher education (54.3% vs. 62.4% in the non-MASLD group).

Health behaviors such as smoking and drinking were more prevalent in the MASLD group. Specifically, the percentage of current smokers and individuals consuming alcohol five or more times per week were both significantly higher in the MASLD group.

In terms of health conditions, the prevalence of hypertension, obesity, and diabetes was markedly higher in the MASLD group. The proportion of individuals with hypertension was 53.9% in the MASLD group compared to only 26.2% in the non-MASLD group. Similarly, the obesity rate was alarmingly higher at 80.0% in the MASLD group versus 6.9% in the non-MASLD group. Diabetes showed a similar pattern, with 22.2% prevalence in the MASLD group against 5.9% in the non-MASLD group.

Overall, the findings from this study underscore significant physiological, sociodemographic, and health behavioral differences between individuals with and without MASLD, highlighting the severe burden of this condition on affected populations. These disparities emphasize the need for targeted interventions and further research into the mechanisms and management of MASLD.

### 3.2 Association between serum cadmium and mercury and MASLD

In the current analysis, we extended our investigation to assess the associations between serum chromium and mercury levels and MASLD, following the observation of no significant differences in serum lead concentrations between MASLD and non-MASLD patients. This examination was crucial to delineate the potential role of other heavy metals in the pathophysiology of MASLD.

Table 2 delineates the association between blood cadmium levels and MASLD across quartiles (Q1 to Q4), with Q1 representing the lowest and Q4 the highest cadmium concentrations. The results, articulated through odds ratios (ORs) in Models 1 through 3, consistently demonstrated a decreasing trend in the odds of MASLD with increasing cadmium levels. Specifically, the ORs indicated a statistically significant inverse association in Model 3, which adjusted comprehensively for demographic, lifestyle, and dietary confounders. This model revealed that individuals in the highest cadmium quartile (Q4) exhibited a notably lower risk of MASLD compared to those in the lowest quartile (Q1).

**Table 2:** Association of blood cadmium and MASLD.

Cadmium	Model 1			Model 2			Model 3		
	OR	95% CI	*p* value	OR	95% CI	*p* value	OR	95% CI	*p* value
Q1	1.000 (Reference)			1.000 (Reference)			1.000 (Reference)		
Q2	0.912	0.814, 1.02	0.111	0.847	0.752, 0.953	0.0062	0.811	0.715, 0.918	0.0011
Q3	0.798	0.708, 0.899	0.0003	0.741	0.649, 0.845	<0.0001	0.638	0.551, 0.738	<0.0001
Q4	0.762	0.681, 0.854	<0.0001	0.732	0.649, 0.825	<0.0001	0.534	0.454, 0.628	<0.0001
P trend	<0.0001			<0.0001			0.0006		

Model 1: unadjusted.

Model 2: adjusted for age, gender, marriage and race.

Model 3: model 2 plus education, RIP, smoke and drink.

Similarly, Table 3 explored the relationship between blood mercury levels and MASLD. The analysis followed a similar quartile-based approach and revealed a consistent inverse pattern across all models. Model 3, adjusting for the broadest range of potential confounders, confirmed a significant reduction in the odds of MASLD among individuals with the highest mercury levels (Q4) compared to those in the lowest quartile (Q1).

**Table 3:** Association of blood Mercury and MASLD.

Mercury	Model 1			Model 2			Model 3		
	OR	95% CI	*p* value	OR	95% CI	*p* value	OR	95% CI	*p* value
Q1	1.000 (Reference)			1.000 (Reference)			1.000 (Reference)		
Q2	1.05	0.926, 1.18	0.465	0.992	0.874, 1.13	0.898	0.991	0.868, 1.13	0.892
Q3	0.923	0.815, 1.05	0.204	0.863	0.762, 0.977	0.021	0.888	0.781, 1.01	0.070
Q4	0.655	0.578, 0.743	<0.0001	0.613	0.540, 0.695	<0.0001	0.648	0.566, 0.743	<0.0001
*P* trend	<0.0001			<0.0001			<0.0001		

Model 1: unadjusted.

Model 2: adjusted for age, gender, marriage and race.

Model 3: model 2 plus education, RIP, smoke and drink.

These findings suggest a paradoxical protective effect of higher serum levels of cadmium and mercury against the risk of MASLD. This inverse relationship, robust across multiple adjusted models, highlights a potentially complex interplay between environmental metal exposures and the pathophysiology of MASLD. Further investigations are necessary to elucidate the mechanisms underlying these associations and to understand the broader implications for public health and preventive measures in populations at risk for MASLD.

### 3.3 Stratified analysis of serum cadmium and mercury levels in relation to MASLD

**Figure 2:**
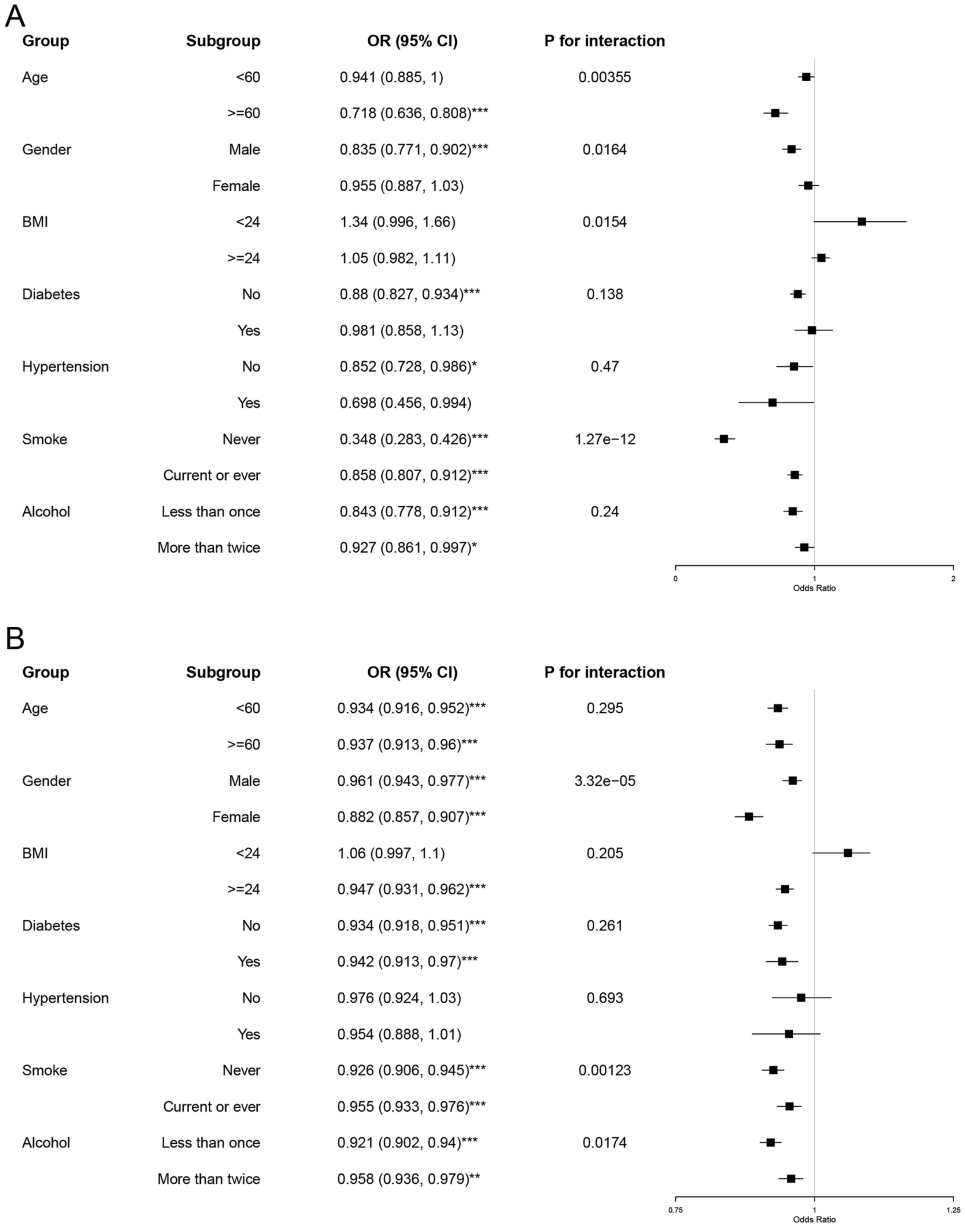
Subgroup and interaction analyses of serum cadmium and mercury levels with MASLD. Forest plots illustrate the results of subgroup analyses and interaction tests. Subgroup analyses evaluate the relationship between serum cadmium **(A)** and mercury levels **(B)** and MASLD among specific demographic and clinical variables (age, gender, BMI, diabetes, hypertension, smoking, and alcohol consumption). Subgroup OR significance is indicated by asterisks (*p < 0.05, **p < 0.001, ***p < 0.0001).

The stratified analysis delineated in Figure 2 aimed to discern the intricate relationship between serum cadmium and mercury levels and the prevalence of MASLD across various demographic and clinical subgroups.

In the case of cadmium, as detailed in Figure 2A, distinct patterns emerged across different subgroups. The odds of developing MASLD were significantly lower in the older cohort (≥60 years) with an odds ratio (OR) of 0.718 (95% CI: 0.636–0.808), compared to the younger cohort (<60 years). This finding was supported by a statistically significant interaction term (P for interaction = 0.00355), suggesting a more pronounced protective effect of higher cadmium levels in older adults. Additionally, males and individuals who never smoked displayed stronger inverse associations with MASLD, with ORs of 0.835 (95% CI: 0.771–0.902) and 0.348 (95% CI: 0.283–0.426), respectively.

For mercury, as shown in Figure 2B, the inverse associations were generally consistent across most subgroups but exhibited a lesser magnitude compared to cadmium. Notably, the protective effect of higher mercury levels was more significant in females (OR = 0.882, 95% CI: 0.857–0.907) and in individuals without diabetes (OR = 0.947, 95% CI: 0.931–0.962). The gender interaction was particularly significant (P for interaction = 3.32e−05), indicating a differential impact of mercury based on gender.

These findings underscore that higher serum levels of cadmium and mercury are associated with a decreased likelihood of MASLD, with variations in the strength of these relationships across different subgroups. The notable interactions pertaining to age with cadmium and gender with mercury highlight the necessity to consider these demographic factors in clinical evaluations and public health interventions aimed at reducing MASLD risk.

### 3.4 Nonlinear relationships between serum mercury and cadmium levels and MASLD

**Figure 3:**
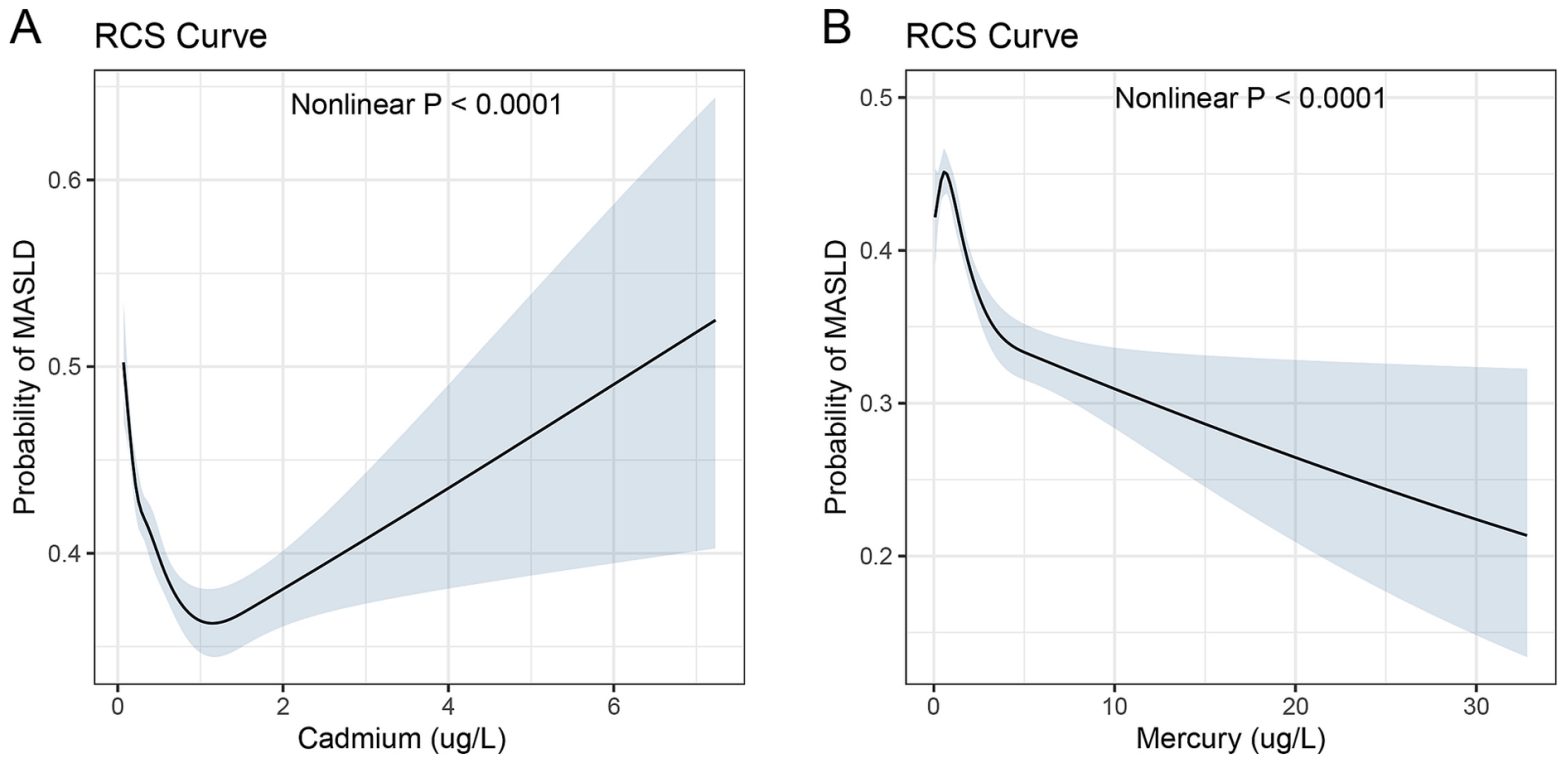
Restricted cubic spline curves for the association between serum heavy metal levels and MASLD. Restricted cubic spline (RCS) curves illustrating the nonlinear associations between serum heavy metal levels (cadmium for panel **A** and mercury for panel **B**) and the probability of MASLD. The shaded areas represent 95% confidence intervals.

In an advanced analytical approach, restricted cubic spline (RCS) curves were employed to rigorously assess the nonlinear relationships between serum levels of mercury and cadmium and the probability of developing MASLD. This analysis, depicted in Figure 3, provides pivotal insights into the dose–response characteristics between these metal exposures and the disease risk.

The RCS curve for cadmium, shown in Figure 3A, demonstrated a nonlinear association with the probability of MASLD. The probability of MASLD increased sharply with initial increases in cadmium levels up to about 1.2 ug/L, followed by a more moderate rise up to 6 ug/L. Although the serum chromium concentration exhibits an upward trajectory beyond 1.2 μg/L, it predominantly remains below 1. Figure 3B demonstrates the RCS curve for mercury. The curve demonstrates a progressive decrease in the probability of MASLD as mercury levels rise from 0 to 30 ug/L, with a notable acceleration in risk observed as levels approach the upper end of this range. The probability of MASLD increases from 0.2 to 0.5 over the observed mercury concentration spectrum. The relationship was statistically significant with a *p*-value of less than 0.0001, indicating a robust nonlinear association.

These RCS analyses underscore the complexity of the relationships between serum levels of mercury and cadmium and MASLD risk. The observed nonlinear patterns highlight critical thresholds beyond which the risk dynamics change significantly, reinforcing the need for targeted preventive strategies based on specific serum concentration ranges.

## 4 Discussion

The present study investigated the associations between serum levels of cadmium and mercury and the risk of metabolic-associated steatohepatitis and liver fibrosis, utilizing data from NHANES from 1999 to 2018. The findings revealed significant inverse relationships between serum cadmium and mercury concentrations and the likelihood of MASLD, with notable variations across demographic and clinical subgroups.

The observed protective effect of higher serum cadmium and mercury levels against MASLD contradicts the conventional understanding of these heavy metals as toxic pollutants. Previous studies have linked cadmium and mercury exposure to various adverse health outcomes, including cardiovascular diseases, kidney dysfunction, and neurodevelopmental disorders (25–30). However, the current study suggests a paradoxical relationship in the context of MASLD, warranting further investigation into the underlying mechanisms. Intriguing research indicating that cadmium treatment alters lipid metabolism within liver cells provide a compelling basis for its potential protective role in MASLD (31). Specifically, the observed decrease in mitochondrial triglycerides and cholesterol may mitigate mitochondrial dysfunction, a key factor in MASLD pathogenesis, while the increase in cytosolic triglycerides and nuclear lipids, including enhanced nuclear phosphatidylcholine synthesis, suggests a reorganization of lipid trafficking that could protect against hepatic steatosis and inflammation. These cellular adaptations imply that cadmium might help maintain lipid homeostasis, potentially reducing the risk of MASLD, despite the well-documented toxic effects of cadmium exposure. This paradoxical role of cadmium highlights the complexity of its impact on liver health and underscores the need for further research to delineate the mechanisms by which cadmium influences liver disease outcomes.

Another study’s findings suggest a nuanced role of cadmium in influencing hepatic lipid metabolism, which may contribute to its potential protective effects against MASLD (32). Despite the upregulation of genes involved in fatty acid synthesis (Fasn and Scd-1) and fatty acid uptake (Fabp1 and Fabp4), cadmium exposure did not lead to insulin resistance or hepatic lipid accumulation, and instead, it improved glucose tolerance. The increase in Lpl gene expression, crucial for lipoprotein lysis, further supports the hypothesis that cadmium might enhance lipid processing and clearance mechanisms. These observations imply that under certain conditions, cadmium could modulate hepatic metabolic pathways to prevent the typical manifestations of MASLD, such as steatosis and insulin resistance, highlighting a complex interaction between cadmium exposure and liver health that warrants deeper investigation.

The stratified analysis revealed that the inverse associations between cadmium and MASLD were more pronounced in older adults, males, and never smokers. These findings suggest that demographic and lifestyle factors may modulate the impact of cadmium exposure on MASLD risk. In the older age groups, the stronger protective effect of cadmium against MASLD may be attributed to the accumulation of cadmium in the body over time (33). As individuals age, they tend to accumulate higher levels of cadmium, which could potentially confer a greater protective effect against the development of MASLD. Another potential explanation for the observed protective effect of cadmium in older individuals could be the age-related changes in metabolic processes (34, 35). As people age, their metabolic responses to environmental toxins may change, potentially modifying how toxins like cadmium are processed and detoxified by the body. These changes may improve the body’s ability to handle these substances without adverse effects, thereby reducing the risk of developing conditions such as MASLD. The gender-specific effects observed in our study, where higher mercury levels were associated with a reduced risk of MASLD more significantly in females. Previous research has indicated that at similar levels of exposure, female tend to accumulate higher levels of mercury in their livers compared to male and are more susceptible to mercury exposure (36). This gender-specific accumulation may have significant implications for liver metabolism in females, potentially altering hepatic functions and increasing the risk of metabolic disorders such as MASLD. Another study confirmed that the health impacts of toxic metals manifest differently between males and females, with variations in prevalence and expression (37). These findings suggest that gender differences in the bioaccumulation and physiological responses to heavy metals are crucial factors that need to be considered in both clinical assessments and public health strategies. The observed enhanced protective effect against metabolic dysfunction-associated steatotic liver disease (MASLD) in individuals who have never smoked is particularly noteworthy, given that smoking is a well-recognized source of cadmium exposure (38, 39). Smoking is a critical factor in the bioaccumulation of cadmium, as evidenced by higher serum cadmium levels in smokers compared to non-smokers (40). This finding is consistent with results from RCS analyses, which reveal that lower cadmium concentrations are linked to a more substantial protective effect against MASLD. Nevertheless, this protective effect diminishes once cadmium concentrations exceed a specific threshold. The interaction noted implies that smoking could intensify the accumulation of cadmium, thereby not only increasing the overall cadmium burden but also potentially altering the threshold at which cadmium begins to have detrimental effects on liver metabolism.

The nonlinear dose–response curves for cadmium and mercury, derived using restricted cubic spline analysis, provide valuable insights into the complex nature of these associations. The sharp increase in MASLD probability at lower cadmium levels, followed by a more gradual rise at higher concentrations, suggests the existence of critical thresholds beyond which the risk dynamics change. Similarly, the accelerated decrease in MASLD risk at higher mercury levels underscores the importance of considering the entire exposure spectrum when assessing health outcomes.

The present study’s strengths include the large, nationally representative sample from NHANES, the comprehensive adjustment for potential confounders, and the advanced analytical approaches employed. However, several limitations should be acknowledged. The cross-sectional design precludes the establishment of causal relationships between serum metal levels and MASLD. Additionally, the use of a single measurement of cadmium and mercury may not fully capture long-term exposure patterns.

Future research should focus on elucidating the biological mechanisms underlying the observed inverse associations between cadmium, mercury, and MASLD. Longitudinal studies with repeated measurements of serum metal levels and assessments of MASLD progression could provide valuable insights into the temporal dynamics of these relationships. Furthermore, investigating the potential interactions between cadmium, mercury, and other environmental pollutants, as well as their combined effects on MASLD risk, could contribute to a more comprehensive understanding of the role of environmental factors in liver disease.

In conclusion, this study reveals significant inverse associations between serum cadmium and mercury levels and the risk of MASLD, with notable variations across demographic and clinical subgroups. These findings challenge the conventional understanding of these heavy metals as universally harmful and highlight the need for a more nuanced approach to assessing their health impacts. Further research is warranted to unravel the complex interplay between environmental exposures and the pathophysiology of MASLD, ultimately informing targeted prevention and intervention strategies.

## Data Availability

The original contributions presented in the study are included in the article/supplementary material, further inquiries can be directed to the corresponding author.
